# Supplementary material for: Structural basis of a two-antibody cocktail exhibiting highly potent and broadly neutralizing activities against SARS-CoV-2 variants including diverse Omicron sublineages
Source: Cell Discov. 2022 Sep 8;8:87. doi: 10.1038/s41421-022-00449-4 (PMC9453709; doi:10.1038/s41421-022-00449-4)
Supplement: Supplementary file 1 — Supplementary Information [file 41421_2022_449_MOESM1_ESM.pdf]

Structural basis of a two-antibody cocktail exhibiting highly potent and broadly neutralizing activities against SARS-CoV-2 variants including diverse Omicron sublineages

Xiaoman Li[1,*], Yongbing Pan[2,*], Qiangling Yin[3,*], Zejun Wang[2], Sisi Shan[4], Laixing Zhang[1], Jinfang Yu[1], Yuanyuan Qu[5], Lina Sun[3], Fang Gui[2], Jia Lu[2], Zhaofei Jing[2], Wei Wu[3], Tao Huang[3], Xuanling Shi[4], Jiandong Li[3], Xinguo Li[2], Dexin Li[3,6], Shiwen Wang[3,6], Maojun Yang[1], Linqi Zhang[4], Kai Duan[2], Mifang Liang[3,6,#], Xiaoming Yang[2,#], Xinquan Wang[1,#]

Affiliations

[1]The Ministry of Education Key Laboratory of Protein Science, Beijing Advanced Innovation Center for Structural Biology, Beijing Frontier Research Center for Biological Structure, School of Life Sciences, Tsinghua University, Beijing, China.

[2]National Engineering Technology Research Center for Combined Vaccines, Wuhan Institute of Biological Products Co. Ltd., Wuhan, Hubei, China.

[3]State Key Laboratory for Molecular Virology and Genetic Engineering, National Institute for Viral Disease Control and Prevention, Chinese Center for Disease Control and Prevention, Beijing, China.

[4]NexVac Research Center, Comprehensive AIDS Research Center, Center for Infectious Disease Research, Department of Basic Medical Sciences, School of Medicine, Tsinghua University, Beijing, China.

[5]Institution of Infectious Diseases, Shenzhen Bay Laboratory, Shenzhen, Guangdong, China.

[6]CDC-WIV Joint Research Center for Emerging Diseases and Biosafety, Wuhan, Hubei, China.

[*]Xiaoman Li, Yongbing Pan, Qiangling Yin contributed equally to this work.

[#]Correspondence Authors:

E-mail address:

liangmf@ivdc.chinacdc.cn (M. Liang); yangxiaoming@sinopharm.com (X. Yang); xinquanwang@mail.tsinghua.edu.cn (X. Wang)

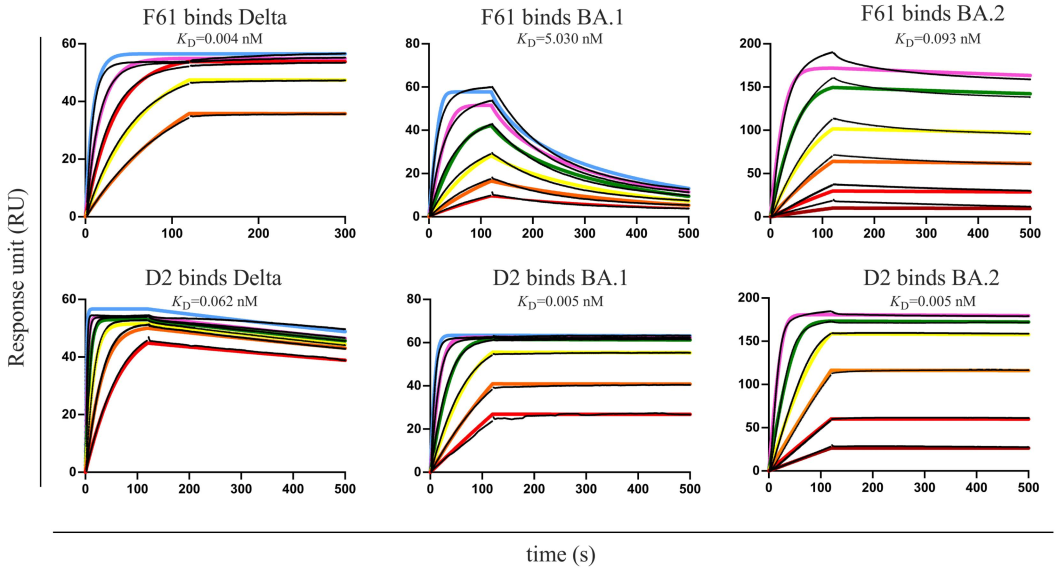

Supplementary Fig. S1. The binding curves between mAbs and SARS-CoV-2 Delta or Omicron RBD measured by SPR. Black lines were the original curves, while colored lines were the fitted curves.

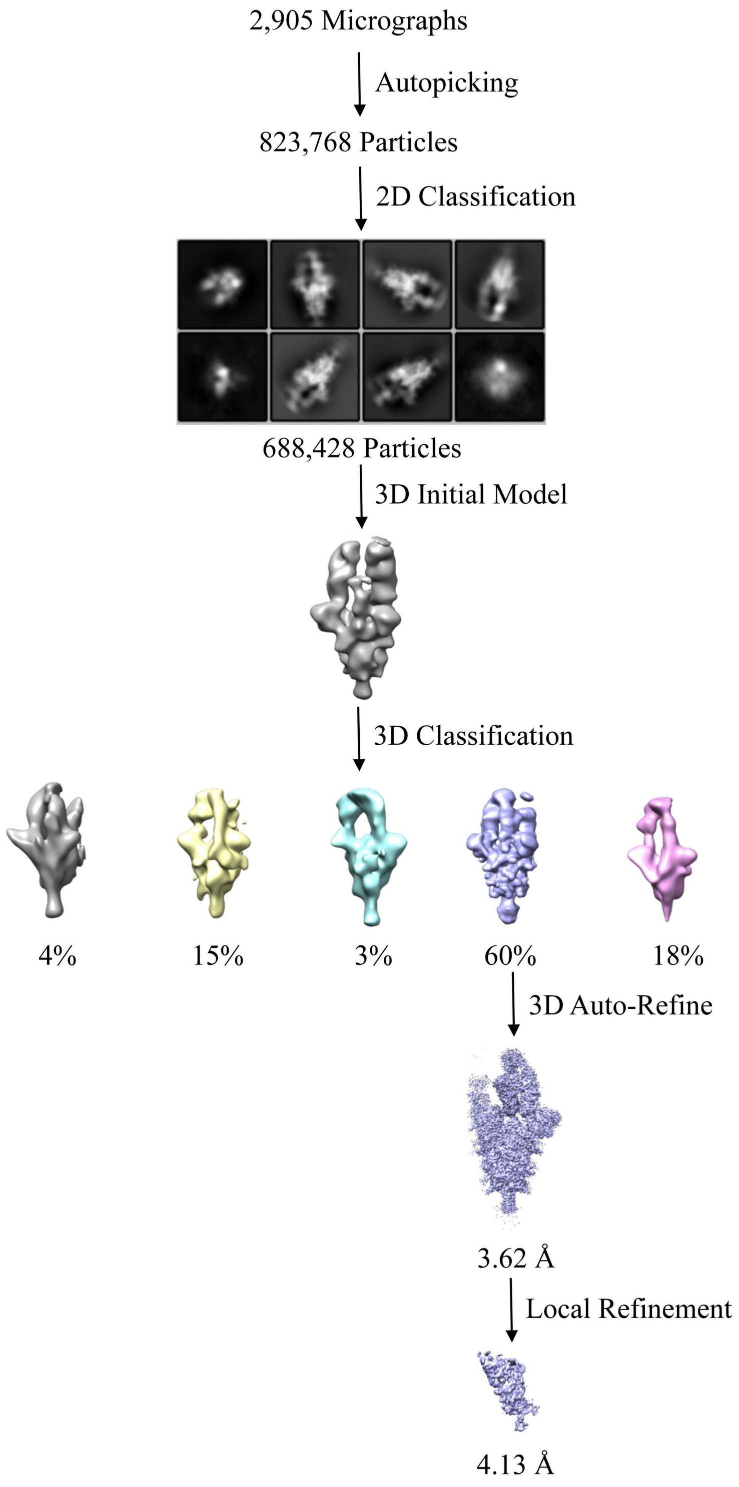

Supplementary Fig. S2. Data processing workflow of the Spike-F61 binary complex Cryo-EM data.

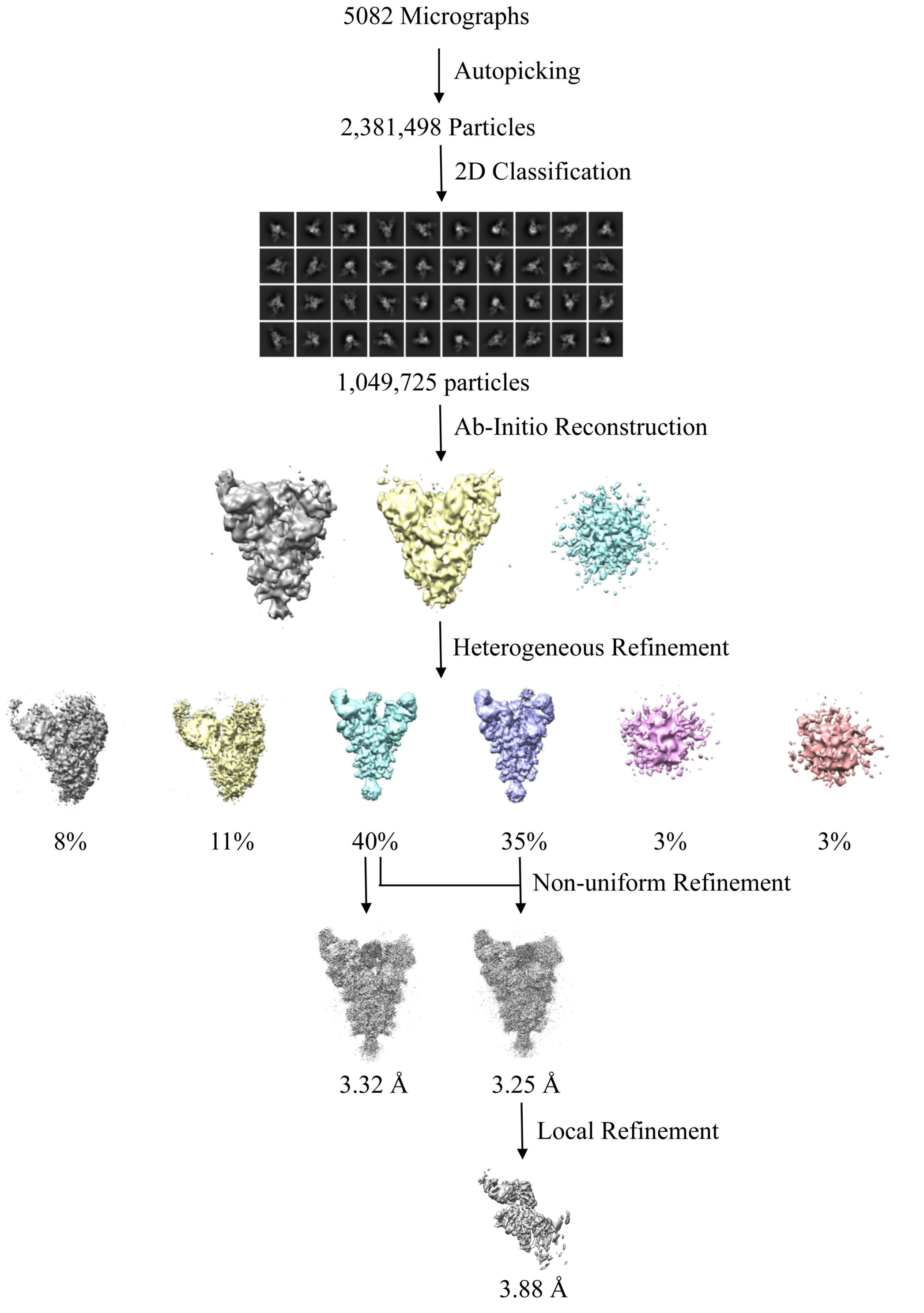

Supplementary Fig. S3. Data processing workflow of the Spike-D2 binary complex Cryo-EM data.

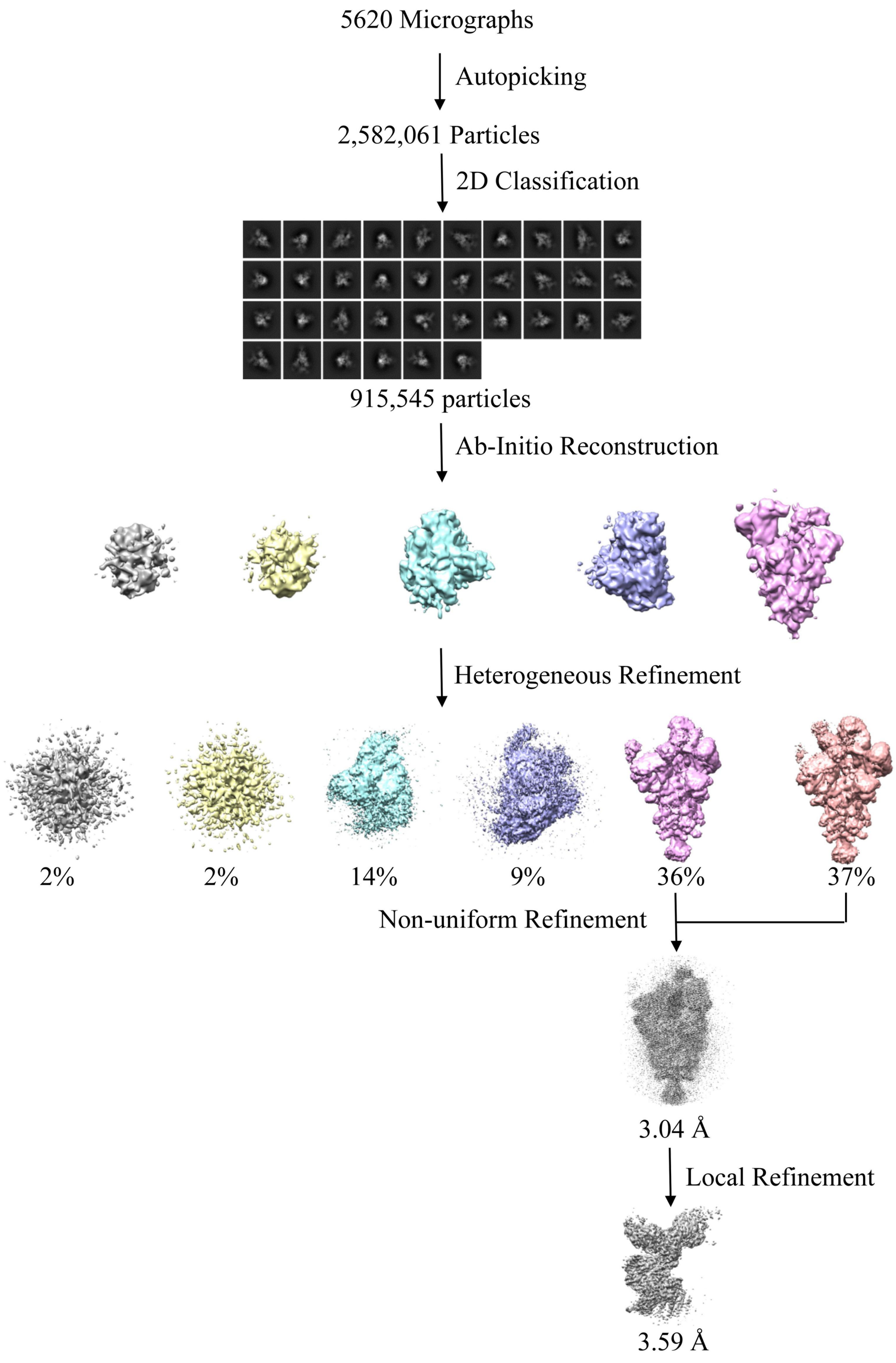

Supplementary Fig. S4. Data processing workflow of the Omicron-Spike-F61-D2 ternary complex Cryo-EM data.

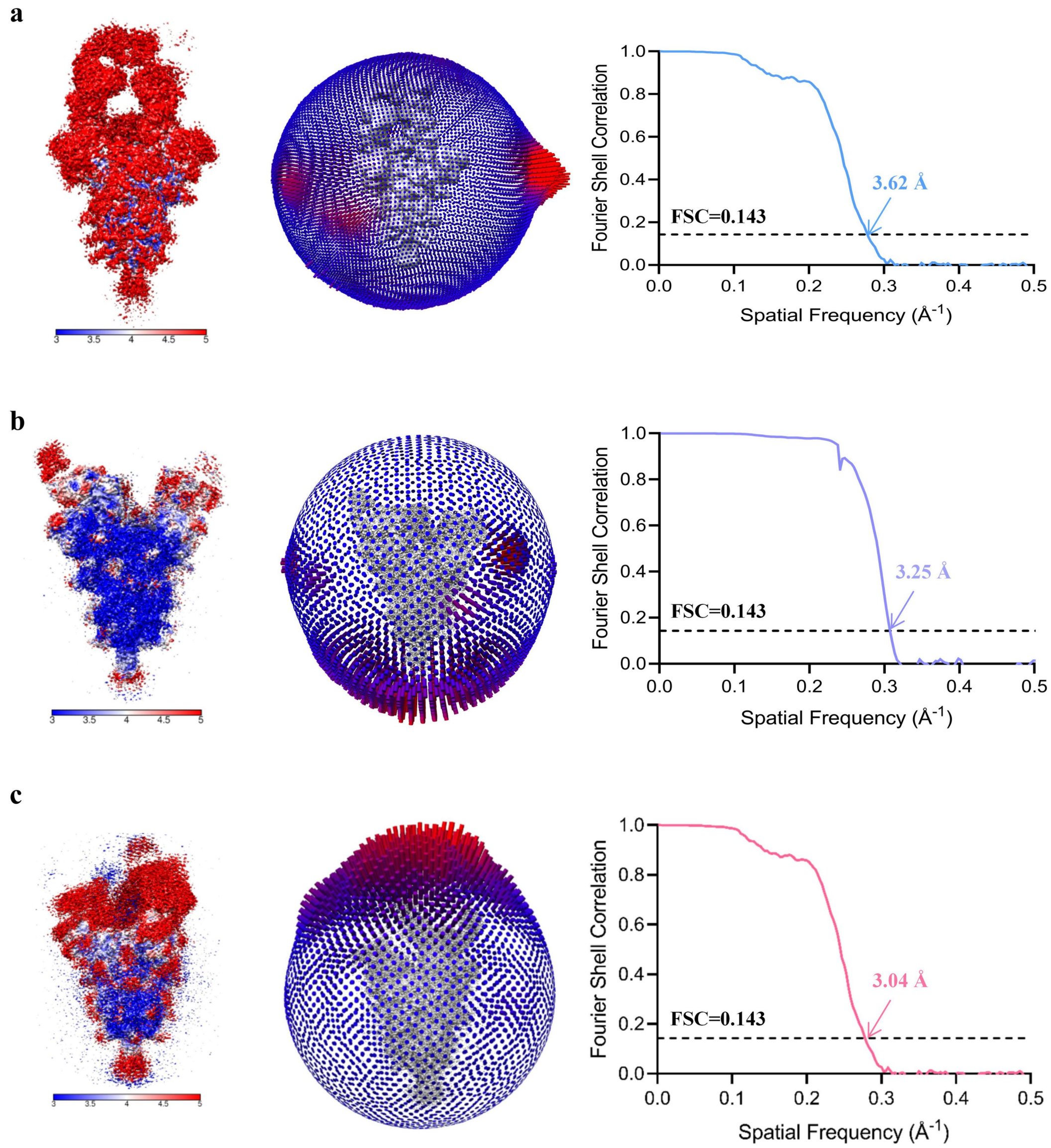

Supplementary Fig. S5. Cryo-EM structure validations. a-c, Local resolution map (left panel), particle orientation distribution (middle panel) and Gold-standard Fourier Shell Correlation (FSC) curves of the final density maps (right panel) of the Spike-F61 complex (**a**), the Spike-D2 complex (**b**) and the Omicron-Spike-F61-D2 complex (**c**). The final resolution of the Spike-F61 complex is 3.62 Å, the final resolution of the Spike-D2 complex is 3.25 Å, and the final resolution of the Omicron-Spike-F61-D2 complex is 3.04 Å.

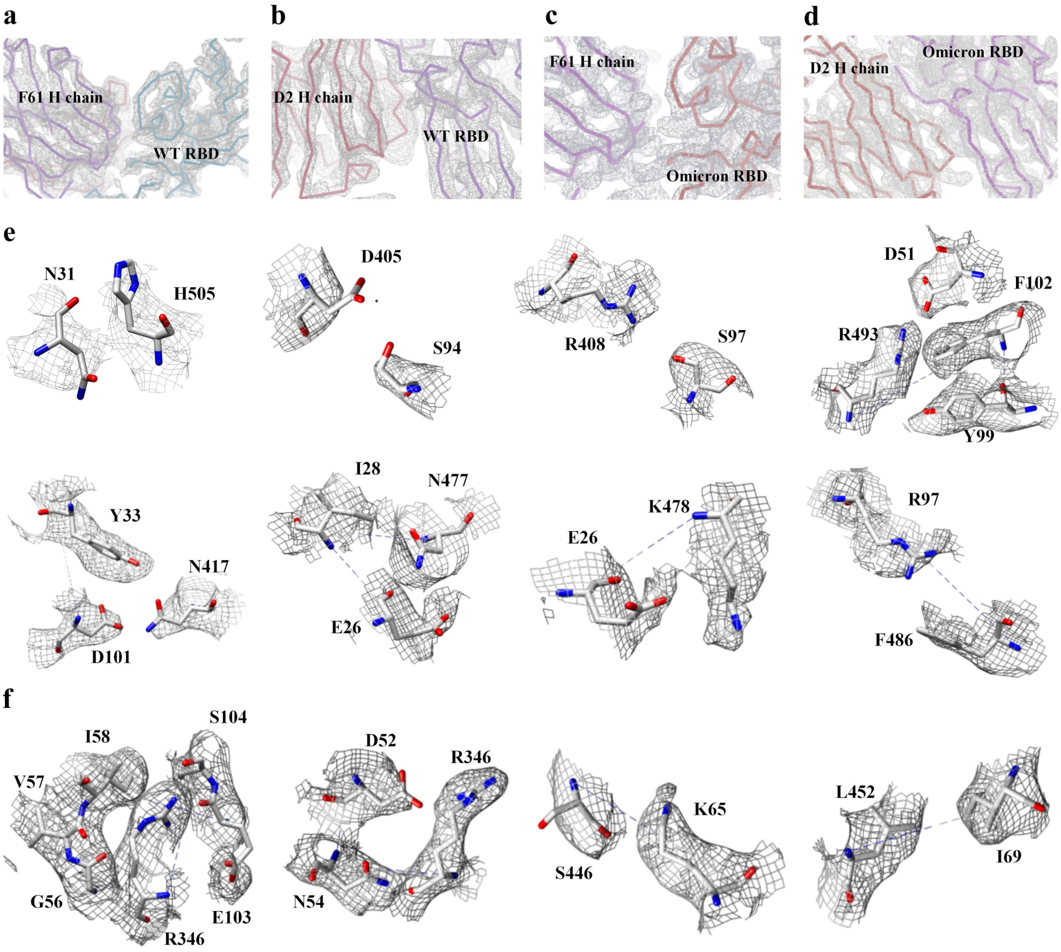

Supplementary Fig. S6. The representative density maps of residues of the Spike-F61, Spike-D2 and Omicron-Spike-F61-D2 complexes. a The representative density maps of the WT-RBD-F61 interface. The map is contoured at 2 RMS to show the density. **b** The representative density maps of the WT-RBD-D2 interface. The map is contoured at 7.2 RMS to show the density. **c** The representative density maps of the Omicron-RBD-F61 interface. The map is contoured at 5.6 RMS to show the density. **d** The representative density maps of the Omicron-RBD-D2 interface. The map is contoured at 5.6 RMS to show the density. **e** The density maps of the Omicron-RBD-F61 interacting residues mentioned in **Fig. 4c**. The map is contoured at 2.2 RMS to show the density. **f** The density maps of the Omicron-RBD-D2 interacting residues mentioned in **Fig. 4d**. The map is contoured at 2.2 RMS to show the density.

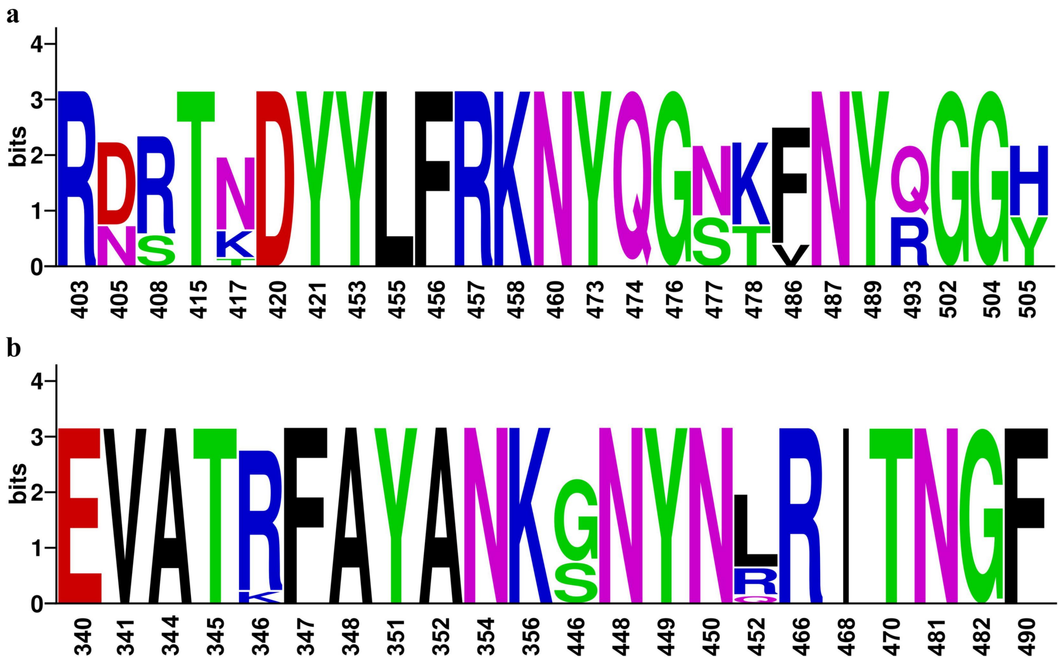

Supplementary Fig. S7. Sequence conservation analysis on antibody epitopes. a-b, Sequence conservation analysis on residues bound by F61 (**a**) and D2 (**b**). These logo plots show the conversation of F61 and D2 epitopes from SARS-CoV-2 WT, Alpha, Beta, Delta, Gamma and Omicron (BA.1, BA.1.1, BA.2, BA.2.12.1, BA.3, BA.4 and BA.5) variants.

a

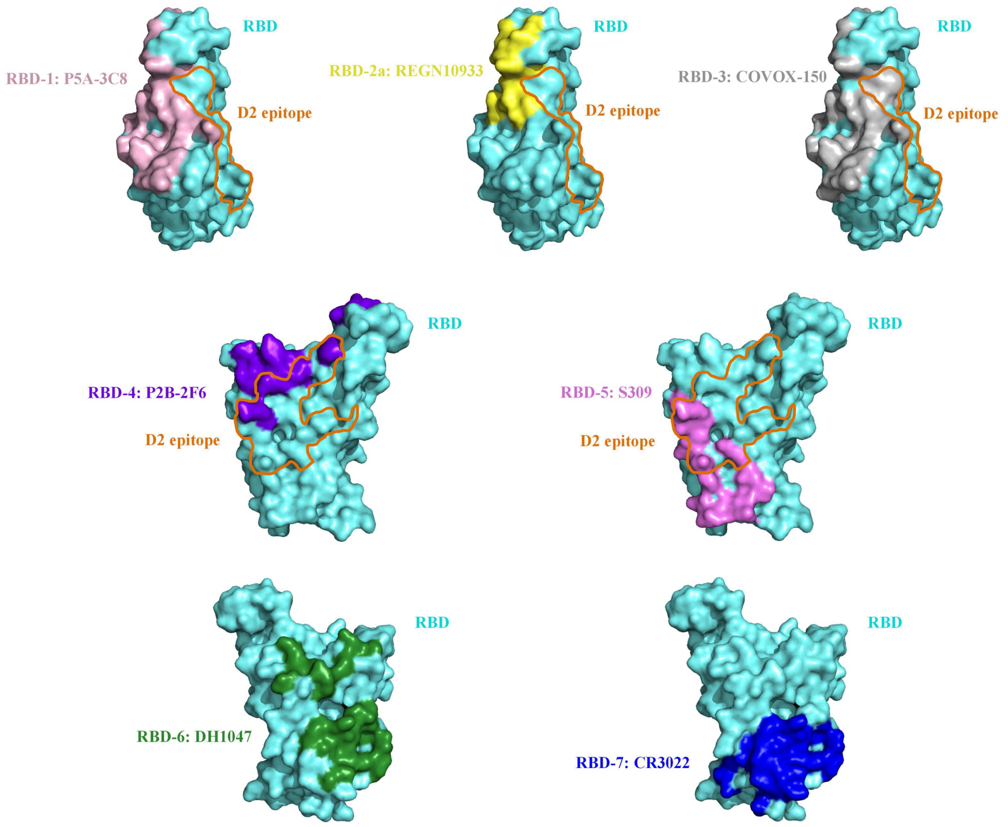

Supplementary Fig. S8. Comparison of D2 epitope with RBD-1 to RBD-7 epitope communities. **a** Structural superposition of Omicron-RBD-D2 and representative antibodies of RBD-1 to RBD-7 (P5A-3C8: 7CHP, REGN10933: 6XDG, COVOX-150: 7BEI, P2B-2F6: 7BWJ, S309: 6WPS, DH1047: 7LD1, CR3022: 6W41) to show the footprints of them. The footprint of D2 is circled by orange line. The footprints of P5A-3C8, REGN10933, COVOX-150, P2B-2F6, S309, DH1047 and CR3022 are represented by pink, yellow, grey, purple, violet, green and blue surfaces, respectively. It was found that D2 epitope largely overlaps with RBD-4 and RBD-5.

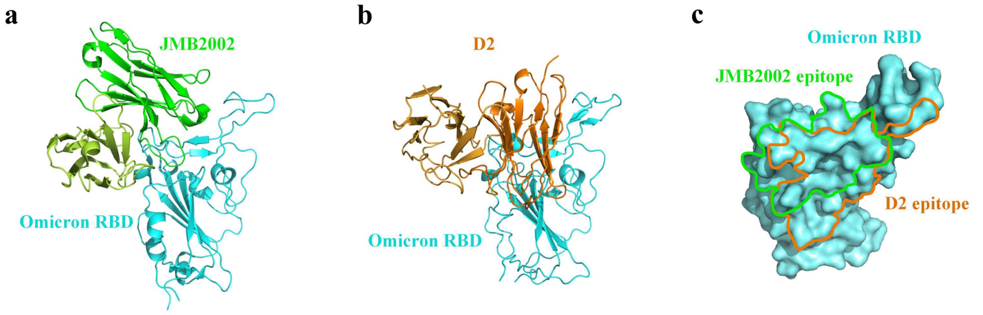

d

JMB2002 epitope

T345 R346 F347 A348 S349 Y351 A352 K440 L441
K444 V445 S446 G447 N448 Y449 N450 Y451 L452
T470 F490 L492 R493 S494 R509

D2 epitope

E340 V341 A344 T345 R346 F347 A348 Y351 A352
N354 K356 S446 N448 Y449 N450 L452 R466 I468
T470 N481 G482 F490

Supplementary Fig. S9. The comparison of Omicron-RBD-D2 and Omicron-RBD-JMB2002 structures. **a** The structure of the Omicron-RBD-JMB2002 (PDB ID: 7WRV) complex. The heavy chain and light chain of JMB2002 are in green and limon, respectively. **b** The structure of the Omicron-RBD-D2 complex. **c** Structural superposition of Omicron-RBD-D2 and Omicron-RBD-JMB2002 (PDB ID: 7WRV) complexes to show the footprints of D2 and JMB2002 on the Omicron-RBD. The footprints of D2 and JMB2002 are circled by orange and green lines, respectively. **d** Omicron-RBD-BA.1 residues recognized by JMB2002 (**left panel**) and D2 (**right panel**). Overlapping residues between the epitopes of D2 and JMB2002 are colored red.

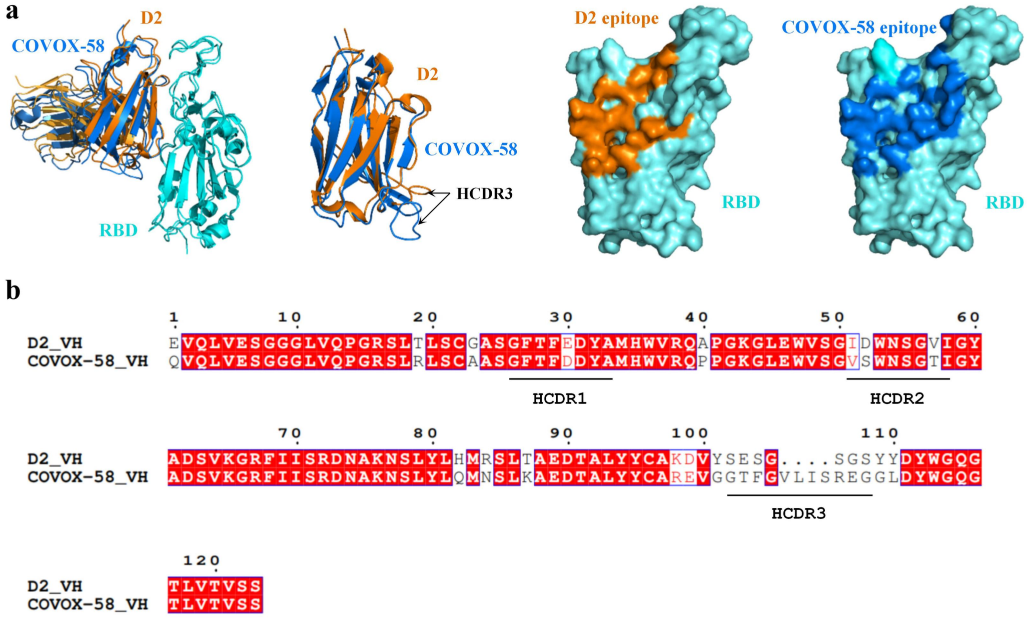

Supplementary Fig. S10. The comparison of RBD-D2 and RBD-COVOX-58 structures and VH sequences. a The structural comparison of the RBD-D2 and RBD-COVOX-58 (PDB ID: 7QNY) complexes. These two structures are superimposed to show a similar binding mode and nearly identical epitope. The structure and epitope of COVOX-58 is shown in marine. HCDR3s of D2 and COVOX-58 are indicated with arrows. **b** Sequence alignment of VH of D2 and COVOX-58.

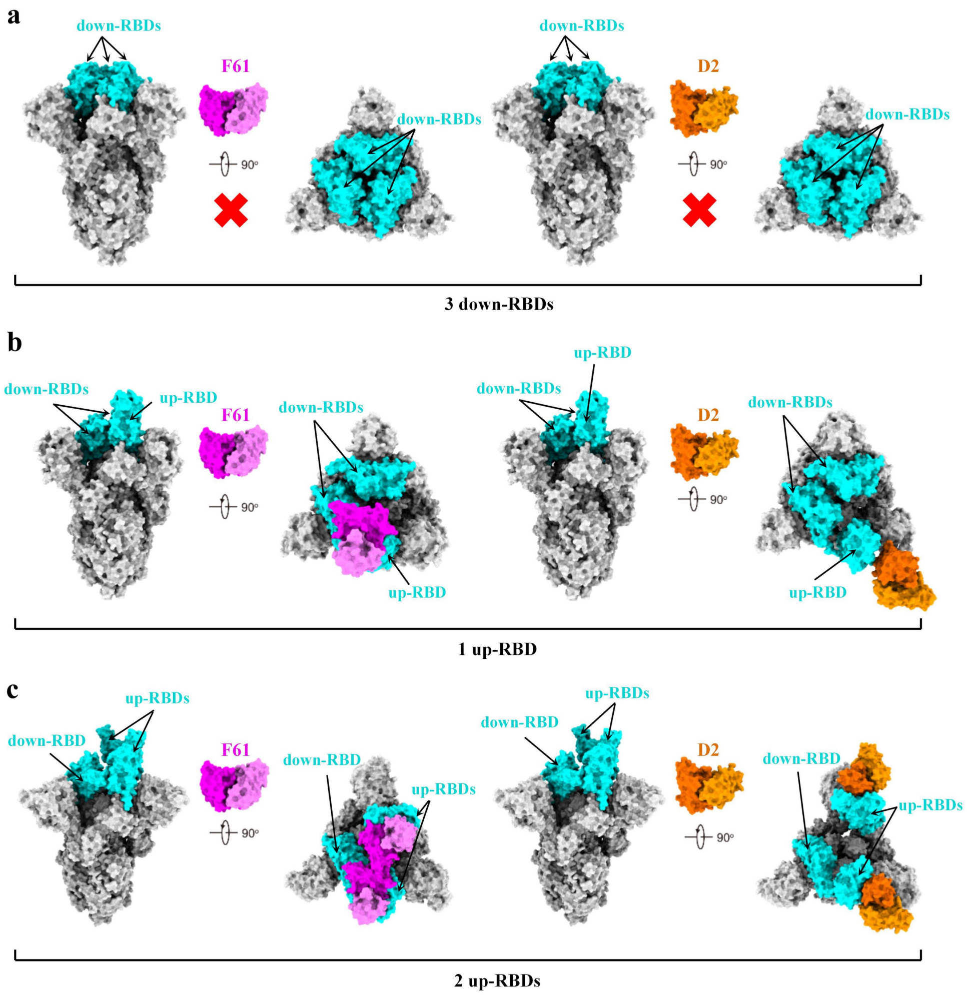

Supplementary Fig. S11. Alignment of the RBD-F61 or RBD-D2 structures onto the spike trimers in the closed form or in the open form with one or two RBDs adopting the up conformation. a-c, Align RBD-F61 or RBD-D2 complexes to spike trimer with three RBDs in the down conformation (**a**, PDB ID: 6VXX), spike trimer with one RBD in the up conformation (**b**, PDB ID: 6VSB) and spike trimer with two RBDs in the up conformation (**c**, PDB ID: 7A93). Red cross means that F61 and D2 cannot bind to down-RBDs in spike trimer due to their clashes with other domains of spike trimer. However, F61 and D2 can bind to up-RBDs of spike trimer with one or two RBDs in the up conformation without clashes, that is to say, F61 and D2 can only bind to up-RBDs.

Supplementary Table S1 Cryo-EM data collection, refinement and validation statistics

	SARS-CoV-2 spike+F61 fab (EMDB-33307) (PDB-7XMX)	SARS-CoV-2 spike+D2 fab (EMDB-33308) (PDB-7XMZ)	SARS-CoV-2 Omicron spike+F61 Fab+D2 fab (EMDB-33434) (PDB-7XST)
**Data collection and processing**			
Magnification	29000×	29000×	29000×
Voltage (kV)	300	300	300
Electron exposure (e–/Å^2)	50	50	50
Defocus range (μm)	-1.2 to -1.5	-1.2 to -1.5	-1.2 to -1.5
Pixel size (Å)	0.97	0.97	0.97
Symmetry imposed	C1	C1	C1
Initial particle images (no.)	~820,000	~2,380,000	~2,580,000
Final particle images (no.)	412558	790425	670525
Map resolution (Å)	3.62	3.25	3.04
FSC threshold	0.143	0.143	0.143
Map resolution range (Å)	3.62-6	3.25-6	3.04-6
**Refinement**			
Initial model used (PDB code)	7KMS	7KMS	7WPF
Model resolution (Å)	3.62	3.25	3.04
FSC threshold	0.143	0.143	0.143
Model resolution range (Å)	3.62	3.25	3.04
Map sharpening *B* factor (Å^2)	-148	-139	-127
Model composition			
Non-hydrogen atoms	28781	29203	33538
Protein residues	3706	3745	4407
Ligands	25	32	31
B factors (Å^2)			
Protein	102.22	75.76	47.63
Ligand	82.91	60.58	57.73
R.m.s. deviations			
Bond lengths (Å)	0.011	0.008	0.008
Bond angles (°)	1.286	1.039	1.370
Validation			
MolProbity score	1.78	1.58	1.78
Clashscore	4.80	5.06	6.78
Poor rotamers (%)	1.34	0.47	0.60
Ramachandran plot			
Favored (%)	93.34	95.48	93.90
Allowed (%)	6.58	4.41	6.08
Disallowed (%)	0.08	0.11	0.02

Supplementary Table S2 The interfacing residues between Fabs and SARS-CoV-2 Omicron Spike

Complex	RBD	Heavy chain	Light chain
**Omicron Spike in complex with F61 fab**	R403		N31, N32
	D405		S94
	R408		S97
	T415	S56	
	N417	Y33, D101	
	D420	S56	
	Y421	Y33, Y52, P53, G54	
	Y453	F102	
	L455	Y33, Y99, F102	
	F456	Y33, Y99	
	R457	P53	
	K458	N30, R31	
	N460	G54	
	Y473	R31	
	Q474	R31	
	G476	I28	
	N477	E26, I28	
	K478	E26	
	F486	R97	
	N487	E26, I27, R97	
	Y489	R97	
	R493	Y99, F102	D51
	G502		N31
	G504		N31
	H505		N31
**Omicron Spike in complex with D2 fab**	E340	W53	
	V341	W53	
	A344	N54	
	T345	E103	
	R346	D52, N54, G56, V57, I58, E103, S104	
	F347	G56	
	A348	N54, S55, G56	
	Y351	I69	
	A352	S55	
	N354	W53, N54, S55	
	K356	W53	
	S446	K65	
	N448	K65	
	Y449	Y60, G66, F68, I69	
	N450	V57, G59	
	L452	I69	
	R466	S55, R72	
	I468	S71, R72, D73	

	T470	H82
	N481	R16
	G482	S17
	F490	I69, R84

A distance cut-off of 4 Å was used.
